# The accordion technique enhances bone regeneration via angiogenesis factor in a rat distraction osteogenesis model

**DOI:** 10.3389/fphys.2023.1259567

**Published:** 2023-09-08

**Authors:** Kai Liu, Sulong Wang, Ainizier Yalikun, Peng Ren, Aihemaitijiang Yusufu

**Affiliations:** Department of Trauma and Microreconstructive Surgery, The First Affiliated Hospital of Xinjiang Medical University, Urumqi, Xinjiang, China

**Keywords:** angiogenesis factor, accordion maneuver, bone regeneration, bone remodeling, distraction osteogenesis

## Abstract

**Objective:** The purpose of this study was to observe the effect of the accordion technique (AT) during the distraction phase on chondrogenesis and bone regeneration in a rat femoral distraction osteogenesis (DO) model, and investigate its potential mechanism for reducing the total treatment time of DO.

**Methods:** Fifty-four male Sprague-Dawley (SD) rats that were specific-pathogen-free (SPF) were subjected to DO surgery on the right femur. The distraction rate was 0.5 mm/day for 10 days, following a latency period of 5 days. Rats were randomly divided into Control (no AT, *n* = 18), Group LA (low amplitude with AT, *n* = 18), and Group HA (high amplitude with AT, *n* = 18) according to different AT protocols in the distraction phase. Rats were respectively euthanized by anesthesia overdose at 2, 4 and 6 weeks of the consolidation phase, and the femurs were harvested. Digital radiography, micro-computed tomography (micro-CT), biomechanical tests, and histomorphological analysis were used to assess the quality of regenerated bone in the distraction area.

**Results:** Digital radiographic, micro-CT, biomechanical tests, and histological analysis revealed an increase in early-stage callus formation (*p* < 0.05) and improved blood supply to the callus tissue in Group LA, as compared to both the Control and Group HA. The enhanced differentiation of fibrous and cartilaginous tissue into bone tissue was also observed in Group LA, leading to improved strength and stiffness (*p* < 0.05) of the regenerated bone at 6 weeks of the consolidation phase. The angiogenic (hypoxia-inducible factor—1α (HIF—1α) and vascular endothelial growth factor (VEGF), *p* < 0.05) and osteogenic (runt-related transcription factor 2 (RUNX2), osteocalcin (OCN) and osteopontin (OPN), *p* < 0.05) biomarkers were higher expressed in Group LA at 2 and 4 weeks of consolidation phase, whereas decreased at 6 weeks of consolidation phase.

**Conclusion:** The application of AT with low amplitude during the distraction phase can enhance chondrogenesis and bone regeneration by activating the angiogenesis factor pathway and upregulating the expression of osteogenic-related biomarkers such as HIF-1α, VEGF, RUNX2, OCN, and OPN.

## Introduction

Distraction osteogenesis (DO) is a bone remodeling technique that stimulates the growth of new bone in the distraction area by gradually pulling apart bone segments, utilizing the principle of tension stress (Ilizarov,1989a; Ilizarov,1989b). Due to the application of a continuous, stable, and moderate distraction force on the callus tissue, DO has demonstrated favorable results in terms of promoting bone union and functional recovery in affected limbs, which has also significantly enhanced the quality of life for millions of patients worldwide who suffer from bone defects (Gubin et al.,2013). Although satisfactory bone and functional results have been received, the risk of postoperative complications resulting from the natural limitations of external fixators, such as pin tract infection, joint stiffness, axial deviation and delayed union, have also been reported (Liu et al.,2020). To address these issues, various tissue engineering strategies have shown promise in promoting bone regeneration and reducing treatment time, including recombinant protein therapy, growth factors, drugs and stem cell therapy (Ai-Aql et al.,2008; Alzahrani et al.,2018). However, most of these interventions are still in the experimental stage and face challenges in terms of cost and complexity for clinical implementation.

DO comprises three distinct phases: the latency phase, which occurs immediately after the osteotomy; the distraction phase, during which the bone segment is gradually pulled apart; and the consolidation phase, which follows the completion of active distraction (Einhorn and Gerstenfeld,2015; Mukhopadhaya and Raj,2017). In the latency phase of DO, the release of interleukins along with growth factors from platelets in the local hematoma promotes the attraction, proliferation, and differentiation of mesenchymal stem cells into osteoblasts and other specialized mesenchymal cells. This process is similar to the inflammatory phase observed in fracture repair. During the distraction phase, the callus tissues undergo gradual elongation as a result of mechanical traction force. Fibroblasts and collagen fibers play a crucial role in the formation of a central fibrous band within the distraction area. Neovascularization takes place between the bundles of collagen fibers, and recruited osteoblasts to align themselves alongside the neovascularization. This alignment ultimately leads to processes such as chondrogenesis, osteogenesis, and primary mineralization (Hamanishi et al.,1994; Aronson,1994). The consolidation phase is the final phase that the callus tissues and cartilage-like tissues undergo mineralization and remodeling. Therefore, interventions during the distraction phase can offer an important strategy to enhance angiogenesis and osteogenesis, ultimately promoting bone regeneration and reducing the duration of the consolidation phase.

The quality of mineralization in the regenerated bone within the distraction area is also influenced by the establishment of an appropriate biomechanical micro-motion environment (Ilizarov,1989b; Carter et al.,1998; Mofid et al.,2002). This environment facilitates the differentiation of stem cells into osteogenic cells (osteogenic differentiation) and promotes the development of new blood vessels (neovascularization), ultimately resulting in improved bone regeneration. The accordion technique (AT) utilizes the principle of tension stress to provide more microvascular tissues for regenerative callus by applying additional biological stress stimulation, which can effectively promote the process of osteogenesis (Mofid et al.,2002). Published studies (Borzunov and Shastov,2019) have reported satisfactory outcomes of AT in the treatment of nonunion at the docking site during ischemic distraction regeneration. However, there is an ongoing debate regarding the ideal protocol for AT, including factors such as the rate, amplitude, and latency, which may introduce some uncertainty regarding the results (Xu et al.,2018). Additionally, there is a risk of pin tract loosening and soft tissue incarceration due to repeated axial compression and distraction of the bone segment. Therefore, the purpose of this study was to observe the effects of AT during the distraction phase on chondrogenesis and bone regeneration in a rat femoral distraction osteogenesis model, and investigate its potential mechanism for reducing the total treatment time of DO.

## Materials and methods

### Animals

Fifty-four male Sprague-Dawley rats weighing 400 ± 30 g were included in this study. These specific-pathogen-free rats were acclimated to a suitable environment with controlled temperature (25°C ± 2°C), humidity (50% ± 5%), and a 12-h day/night cycle. They were provided free access to food and water, ensuring their stable condition throughout the experiment. All experimental procedures were subjected to review and approval by the Ethics Committee of our institute (Approval number: IACUC-20200318-82). The study was conducted following the ethical standards for animal research in China.

### Surgical technique

The rats were anesthetized with an intraperitoneal injection of 2% pentobarbital sodium (3 mg/100 g, Sigma, St. Louis, MO, US). A 30 mm longitudinal incision was performed at the lateral thigh to expose the middle part of the femur. Four Kirschner wires (1.2 mm) were respectively inserted at the proximal and distal of the femur to install a custom unilateral distraction external fixator (accuracy of 0.125 mm, Supplementary Figure S1). A mid-diaphysis transverse osteotomy was conducted, and the broken bone chips were washed away with 0.9% saline, followed by the incision suture. After surgery, rats were single housed for the remainder of the study with pin tract care. Benzylpenicillin (200,000 IU/kg, Shanghai Baite Pharmaceutical Co., Ltd., Shanghai, China) was injected subcutaneously for 3 postoperative days to prevent infection.

### AT protocol

DO was comprised of three phases: latency phase for 5 days, distraction phase for 10 days, and consolidation phase for 6 weeks. In the Group Control (*n* = 18), bone distraction was performed at a rate of 0.25 mm/12 h for 10 days without utilizing AT. The AT protocol for Group LA (low amplitude with AT, *n* = 18) consisted of a distraction of 0.75 mm followed by compression of 0.25 mm with a 12-h interval, repeated for 10 days. For Group HA (high amplitude with AT, *n* = 18), the AT protocol involved a distraction of 1 mm followed by compression of 0.5 mm with a 12-h interval, repeated for 10 days (Table 1). At 2, 4 and 6 weeks of the consolidation phase, rats were randomly selected and sequentially euthanized by anesthesia overdose to harvest the femurs (Table 2).

**TABLE 1:** DO using AT protocol by unilateral external fixator.

Time	Control (*n* = 18)	LA (*n* = 18)	HA (*n* = 18)
8:00 a.m.	Distraction0.25 mm	Distraction0.5 mm	Distraction0.75 mm
12:00 a.m.	-	Compression0.25 mm	Compression0.5 mm
4:00 p.m.	-	Distraction0.5 mm	Distraction0.75 mm
8:00 p.m.	Distraction0.25 mm	Compression0.25 mm	Compression0.5 mm

AT, accordion technique; DO, distraction osteogenesis; HA, group high amplitude; LA, group low amplitude.

**TABLE 2:** Examination assignment of collected samples in 2-week, 4-week and 6-week of consolidation phase.

Time point	Method	Control (*n* = 18)	LA (*n* = 18)	HA (*n* = 18)
2-week	Digital radiographic/Micro-CT	3/-	3/-	3/-
	TPBT	-	-	-
	Histomorphological analysis	5	5	5
4-week	Digital radiographic/Micro-CT	3/-	3/-	3/-
	TPBT	-	-	-
	Histomorphological analysis	5	5	5
6-week	Digital radiographic/Micro-CT	3/5	3/5	3/5
	TPBT	3	3	3
	Histomorphological analysis	5	5	5

HA, group high amplitude; LA, group low amplitude; Micro-CT, Micro-computed tomography; TPBT, Three-point bending test.

### Bone formation outcomes *in vivo*

Under brief anesthesia using isoflurane, a digital radiographic test using the HF400VA system (MIKASA, Tokyo, Japan) was used every 2 weeks on the right femur of each rat to assess the regenerated bone tissue in the distraction area. At 6 weeks of the consolidation phase, the collected femurs were fixed in a 4% paraformaldehyde solution for 48 h at room temperature for micro-computed tomography (micro-CT) scan. After fixation, the femurs were rinsed three times (5 min per rinse) with phosphate buffer saline and subsequently transferred to a 75% ethanol solution for storage at 4°C until further use. The femurs were rehydrated in a 0.9% saline solution overnight and then wrapped in moistened cotton gauze soaked in 0.9% saline before scanning. The collected femurs (*n* = 5/group) were then scanned using micro-CT with a voxel size of 18 μm, 70 kV, 385 µA for 200 ms (SkyScan, Bruker, Rheinstetten, Germany). Three-dimensional algorithms provided by SkyScan CTAn software were employed to analyze these images, and the 5 mm distraction area was defined as the region of interest (ROI). Quantitative analysis of callus formation within the ROIs included measurements such as Bone Mineral Density (BMD), Bone Volume (BV), Total Volume (TV), Percentage of bone volume to total volume (BV/TV), Trabecular Number (Tb. N), Trabecular Thickness (Tb. Th), and Trabecular Separation (Tb. Sp).

### Biomechanical test

Three-point bending test was conducted at room temperature to assess the mechanical properties of the collected fresh femurs (*n* = 3/group). The testing was carried out using a three-point bending system (RGM-3005T, Shenzhen Reger Instrument Co., Ltd., China). During the test, the femoral long axis was aligned perpendicular to the blades, with a span of 18 mm, and the distraction area of the specimens was subjected to a constant anteroposterior load applied at a rate of 0.5 mm/min until failure. The non-normalized stiffness (N/mm), ultimate load (N), and energy to failure (N/mm^2^) were recorded and analyzed using the algorithm specific to the three-point bending test (Leppanen et al.,2006). The built-in software (REGER, Shenzhen Reger Instrument Co., Ltd., China) was utilized for data analysis.

### Histomorphometric evaluation

After radiological evaluation and micro-CT scanning, five specimens from each group were randomly selected at each time point and subjected to decalcification in a 10% ethylenediaminetetraacetic acid (EDTA) solution for 4 weeks. Subsequently, the samples underwent sequential processes of dehydration, transparency, and paraffin embedding. Using a microtome (RM2135, Leica, Wetzlar, Germany), sagittal sections (5 μm thick) of ROI were cut. Hematoxylin and eosin (H&E) staining (G1004, Wuhan Servicebio Technology Co., Ltd., Wuhan, China), Safranin O-Fast Green staining (G1053, Wuhan Servicebio Technology Co., Ltd., Wuhan, China), and Masson Trichrome staining (G1006, Wuhan Servicebio Technology Co., Ltd., Wuhan, China) were performed for histological analysis, according to the standard procedures. Safranin O-Fast Green staining was employed to distinguish the osteogenesis and cartilage, indicating that cartilage was stained red and subchondral bone was stained green. Collagen fibers, mucus and cartilage were stained blue in Masson Trichrome staining, while cytoplasm, muscle, cellulose and glial were stained red.

Immunohistochemistry staining involved the use of specific antibodies, including anti-hypoxia-inducible factor (HIF)-1α (ab51608, 1: 100, Abcam, Cambridge, UK), anti-vascular endothelial growth factor (VEGF) (sc57496, 1:100, Santa Cruz, CA, US), anti-runt-related transcription factor 2 (RUNX2) (sc390715, 1:100, Santa Cruz, CA, US), anti-osteocalcin (OCN) (ab93876, 1:100, Abcam, Cambridge, UK), and anti-osteopontin (OPN) (ab166709, 1:100, Abcam, Cambridge, UK). After the samples were incubated in a secondary antibody (ZSGB-BIO, Beijing, China) at 37°C for an hour, signal detection was performed using a horseradish peroxidase-streptavidin system (ZSGB-BIO, ZLI-9019, Beijing, China) followed by counterstaining with hematoxylin. The samples were examined and imaged using a high-quality microscope (Olympus CX43, Olympus Co., Ltd., Tokyo, Japan), and Six random fields of ROI from each section were analyzed in a blinded manner under a magnification of ×200 using DP26 imaging software (OLYMPUS, Japan). The integrated optical density in the positively stained area or cells (brown) on the section was semi-quantitatively analyzed using Image Pro Plus 6.0 software (Media Cybernetics, Inc., Rockville, MD, US).

### Statistical analysis

The quantitative variables were expressed as mean ± SD. The normality of the data was evaluated by the Shapiro–Wilk test. The Mann–Whitney *U*-test was utilized to assess statistical differences when data was skewed. One-way analysis of variance (ANOVA) with *post hoc* pair-wise comparisons was used to determine differences when data was not skewed. All analyses and graphs were performed by GraphPad Prism 9.0 software (San Diego, CA, US). Statistical significance was *p* < 0.05.

## Results

**FIGURE 1:**
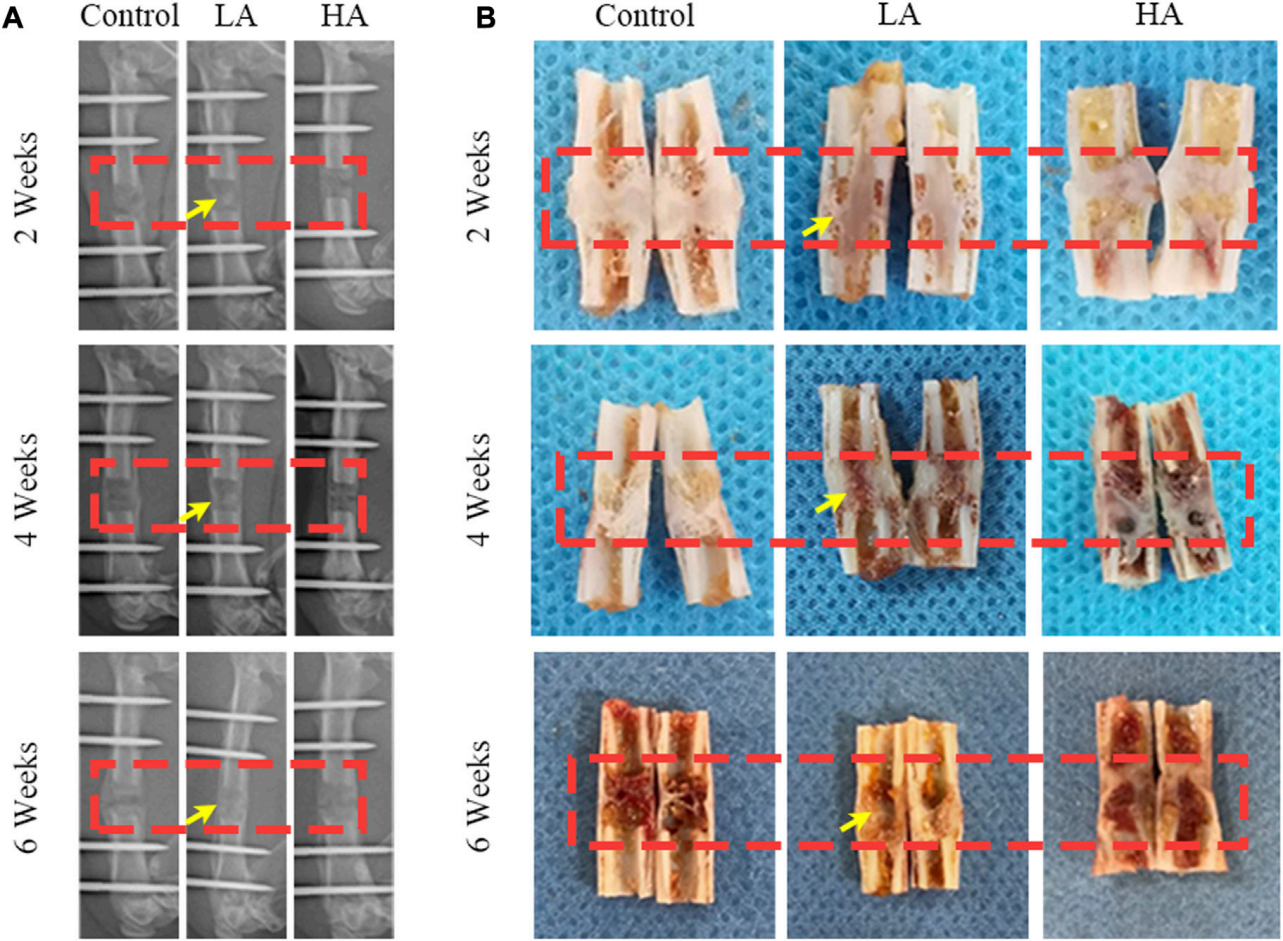
The general images and X-ray images of ROI until the 6-week consolidation phase terminated. **(A)** X-ray of the distraction area, the regenerated bone of the distraction area in Group LA (red areas) was markedly increased (yellow arrows) at the consolidation phase. **(B)** The general appearance of the distraction area (red areas) showed that recanalization of the medullary cavity (yellow arrows) was received in Group LA at 6 weeks of consolidation.

**FIGURE 2:**
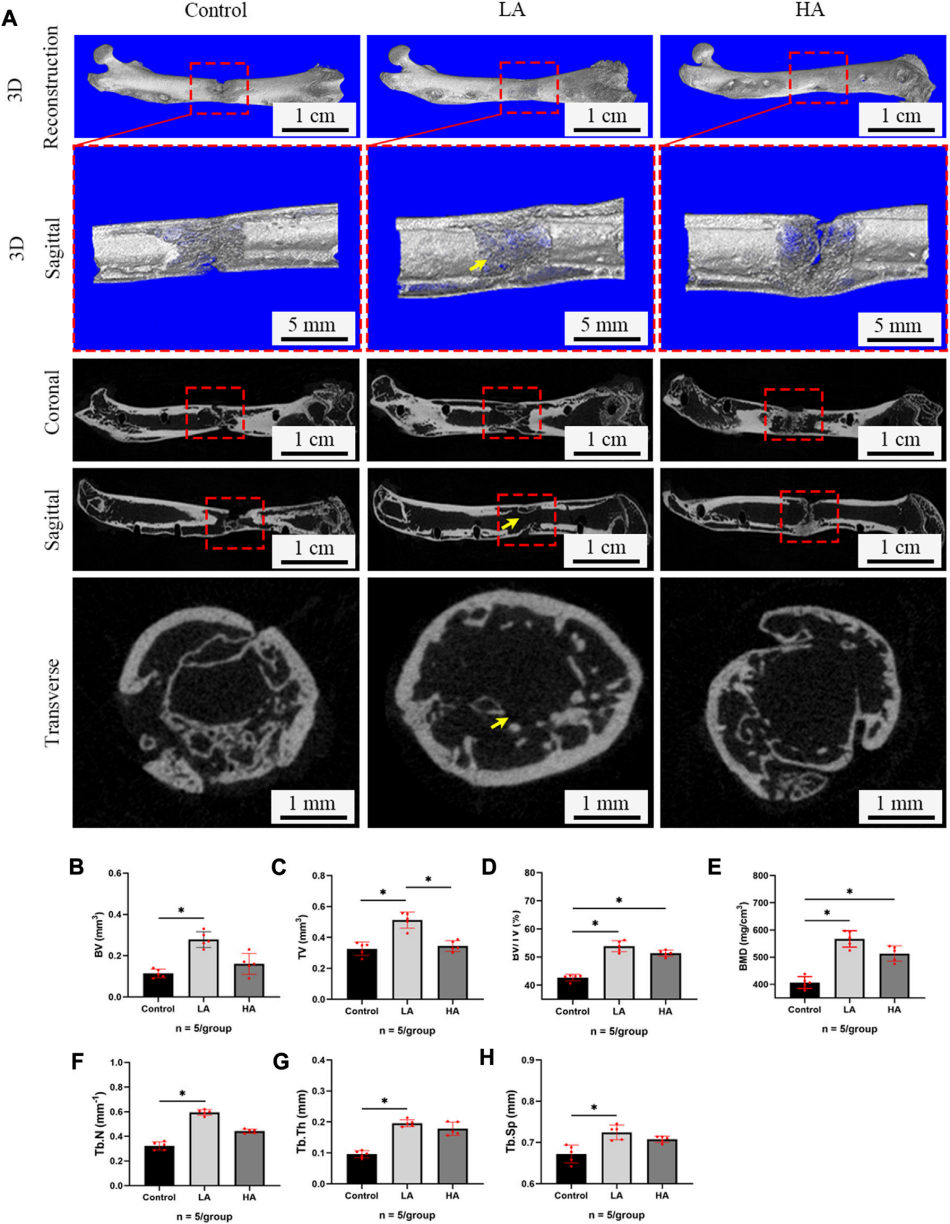
Micro-CT evaluation of ROI at 6 weeks of consolidation phase. **(A)** Representative 3D micro-CT images of the distraction area (red areas) at 6 weeks of the consolidation phase showed that recanalization of the medullary cavity (yellow arrows) was received in Group LA. However, narrow gaps in the distraction area were observed in the Control and Group HA. **(B–H)** Quantitative evaluation of the values of BMD, BV, TV, BV/TV, Tb. N, Tb. Th, Tb. Th, and Tb. Sp in Group LA and Group HA were significantly higher than the Control (*p < 0.05; ns, *p* > 0.05).

The mineralization progression of ROI was assessed using digital radiography, general observation, and micro-CT scanning in the three groups (Figures 1, 2). In Group LA, the status of bone mineralization and continuity of the marrow cavity was superior to the other two groups, as evident from the micro-CT images taken at the 6 weeks of consolidation phase. Additionally, the values of BMD (Group LA vs. Control, 567.1 ± 30.37 vs. 406.4 ± 22.11 mg/cm^3^, *p* < 0.001), BV (0.29 ± 0.04 vs. 0.11 ± 0.02 mm^3^, *p* = 0.004), TV (0.51 ± 0.05 vs. 0.32 ± 0.04 mm^3^, *p* = 0.014), BV/TV (53.87% ± 1.96% vs. 42.71% ± 1.15%, *p* = 0.003), Tb. N (0.59 ± 0.02 vs. 0.32 ± 0.03 mm^-1^, *p* = 0.001), Tb. Th (0.19 ± 0.01 vs. 0.09 ± 0.01 mm, *p* = 0.008), and Tb. Sp (0.72 ± 0.01 vs. 0.67 ± 0.02 mm, *p* = 0.006) in Group LA were significantly higher compared to the Control (Figure 2). Although the above values of Group HA were higher than those in the Control, there was no statistically significant difference were observed (*p* > 0.05).

**FIGURE 3:**
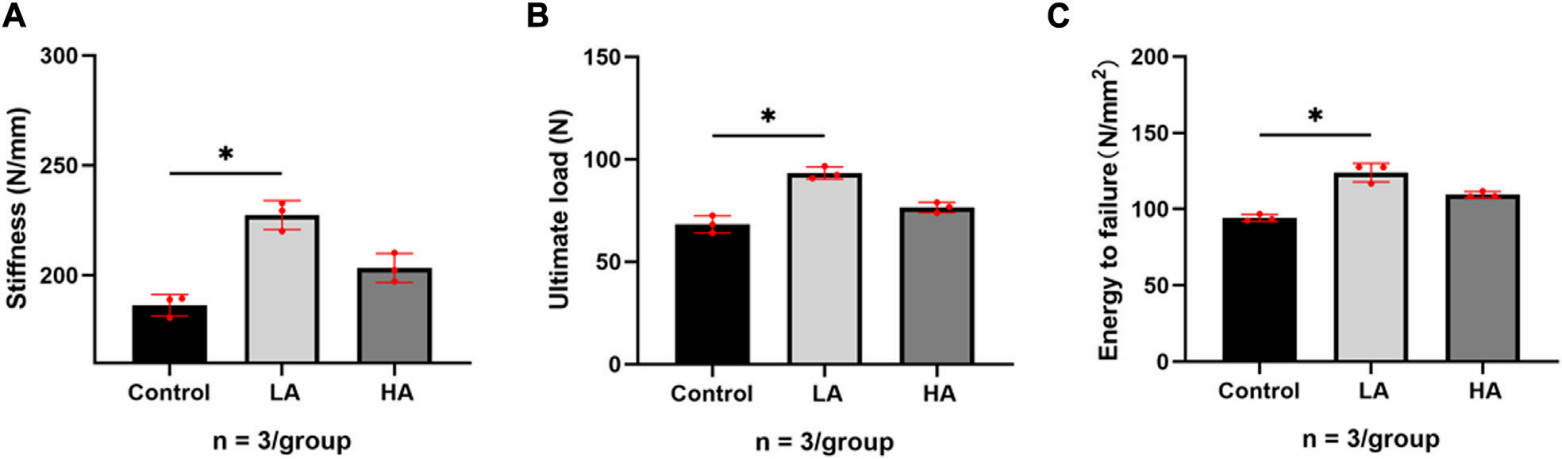
Results of mechanical values of ROI after 6 weeks of consolidation. **(A–C)** The non-normalized stiffness (N/mm), ultimate load (N), and energy to failure (N/mm2) in Group LA and Group HA were significantly better than those in Control (*p < 0.05).

At the 6 weeks of the consolidation phase, a three-point bending test was conducted to assess the mechanical properties of the collected femurs. The non-normalized stiffness (Group LA vs. Control, 227.4 ± 6.60 vs. 186.3 ± 4.91 N/mm, *p* = 0.021), ultimate load (93.29 ± 3.01 vs. 68.29 ± 4.20 N, *p* = 0.039), and energy to failure (123.9 ± 6.15 vs. 94.16 ± 2.27 mg/cm^3^, *p* = 0.025) of Group LA were found to be significantly superior to the Control (Figure 3). The Group HA showed better mechanical properties than the Control, however, no significant difference was observed relative to these two groups (*p* > 0.05).

**FIGURE 4:**
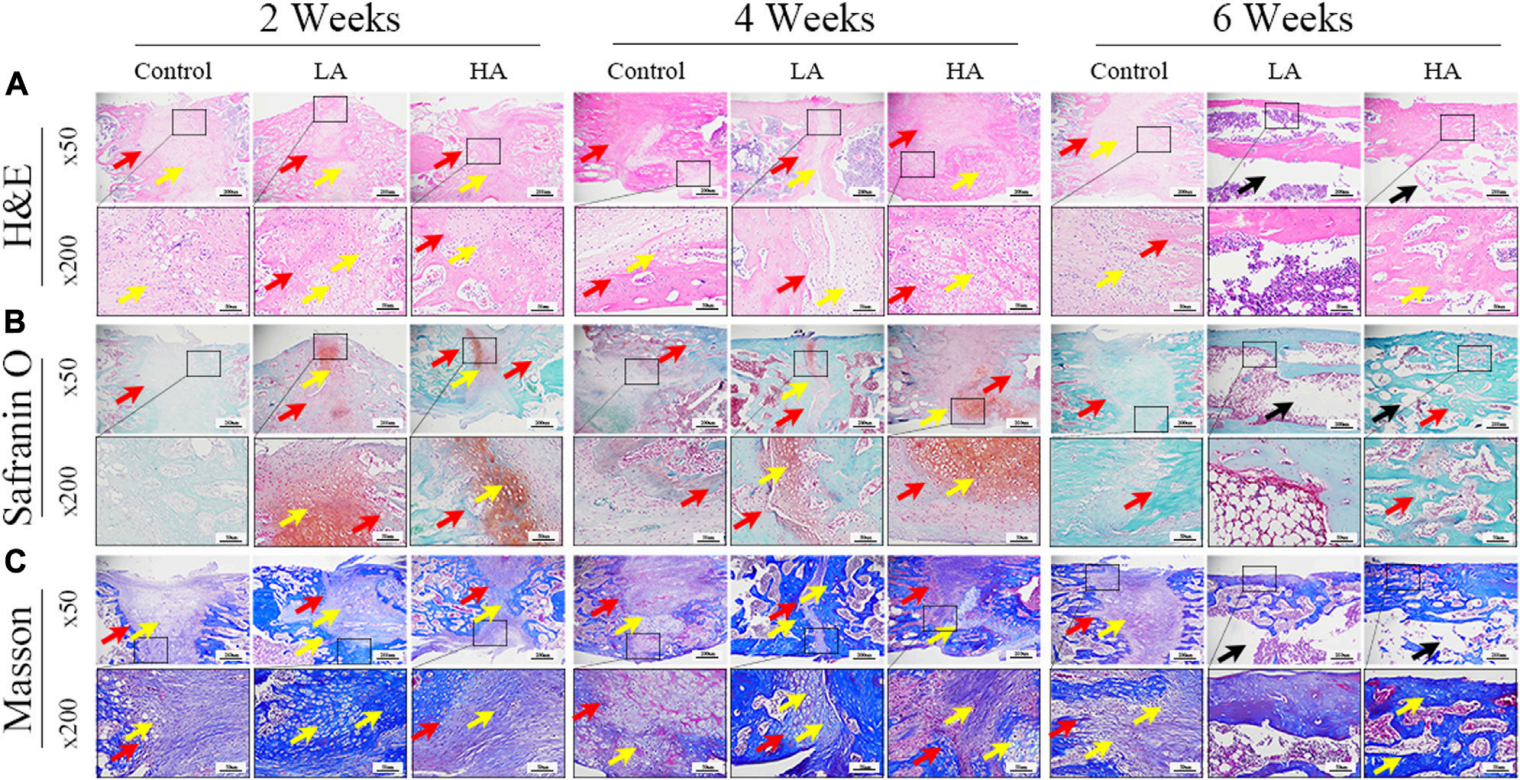
The histomorphological analysis of ROI (low-magnification × 50, and high-magnification × 200) during the consolidation phase. **(A)** H&E staining revealed more microvessels, bone trabeculae, and callus formation (yellow arrows) in Group LA compared to the other two groups at 2 and 4 weeks of the consolidation phase. The two osteogenic fronts (red arrows) originated at the proximal and distal ends of the distraction area and gradually progressed towards the center until the medullary cavity was completely recanalized. By 6 weeks, the recanalization of the medullary cavity (black arrows) in the distraction area of Group LA was achieved. In contrast, both the Control and Group HA still exhibited a significant amount of callus and immature cartilage-like tissue (yellow arrows), with no recanalization of the medullary cavity. **(B)** Safranin O-Fast Green staining demonstrated increased chondrogenesis (red staining indicated by yellow arrows, primarily composed of prechondrocytes) and bone formation (green staining) in Group LA at 2 and 4 weeks of the consolidation phase. **(C)** Masson trichrome staining indicated enhanced chondrogenesis (blue collagen fibers and cartilage, yellow arrows) and bone regeneration in Group LA at 2 and 4 weeks of the consolidation phase. Moreover, these cartilage-like tissues gradually transformed into mature bone tissue with medullary recanalization (black arrows) by 6 weeks of the consolidation phase.

The H&E staining, Safranin O staining, and Masson trichrome staining results of the samples collected at 2, 4, and 6 weeks of consolidation phase were depicted in Figure 4. The ×50 and ×200 magnification images of ROI provided visual confirmation of a significant increase in the area of regenerative callus tissue over time in both Group LA and Group HA, as compared to the Control group. Moreover, Group LA demonstrated better chondrogenesis and bone regeneration in the distraction area when compared to Group HA. At the 6 weeks of the consolidation phase, partial recanalization of the medullary cavity was observed in Group LA, which aligned with the findings from micro-CT.

**FIGURE 5:**
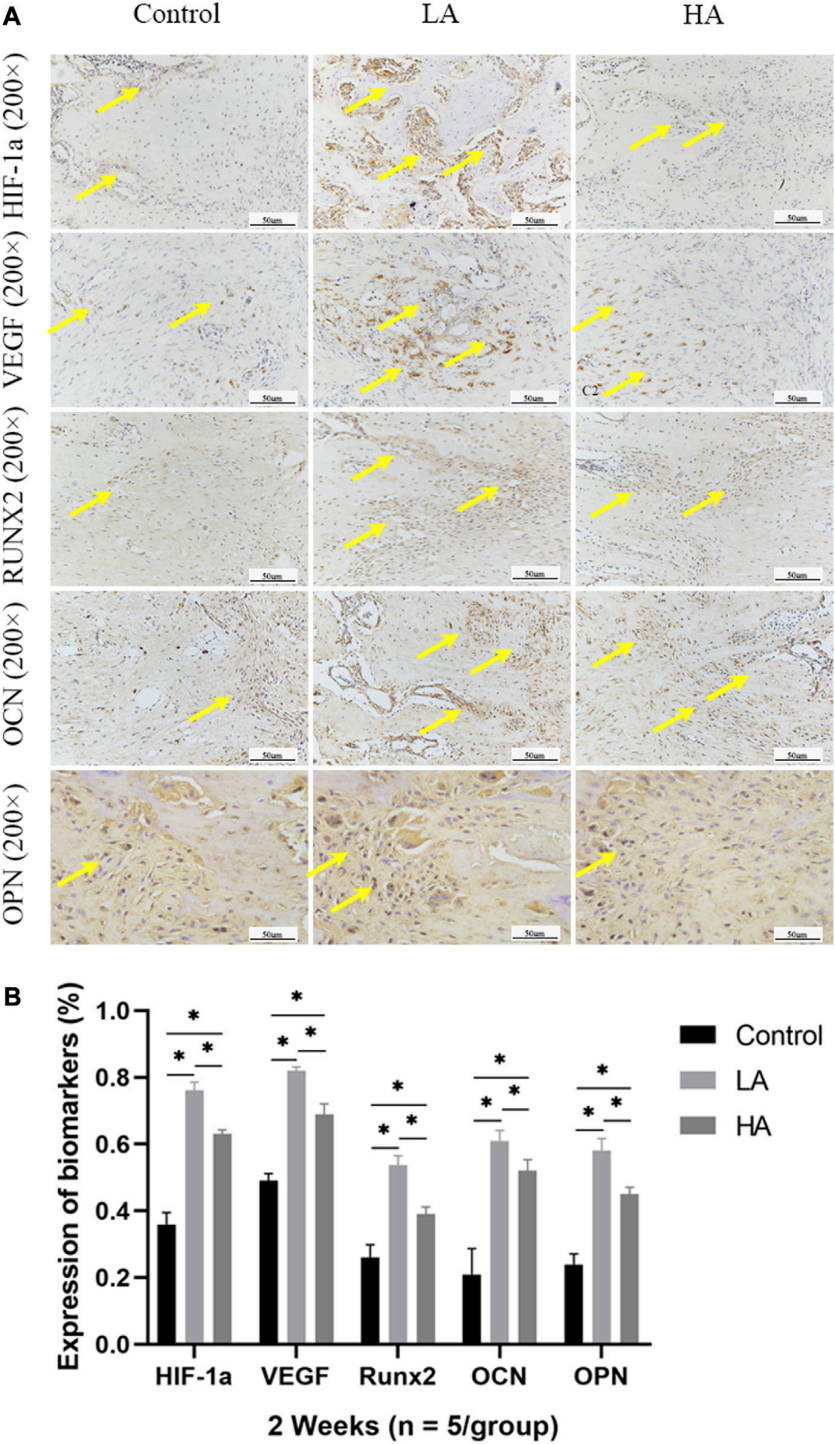
Immunohistochemistry images (high-magnification 200 ×) in the three groups at 2 weeks of consolidation phase **(A)** In Group LA, the positive staining area or cells (yellow arrows) were predominantly localized in the central region of the distraction area, whereas in Group HA and the Control group, they were mainly observed at the edge of the distraction area. **(B)** At the 2 weeks of consolidation phase, the semi-quantitative measurements demonstrated a significant upregulation of HIF-1α, VEGF, RUNX2, OCN, and OPN expression in Group LA, compared to Group HA and the Control (*p < 0.05).

**FIGURE 6:**
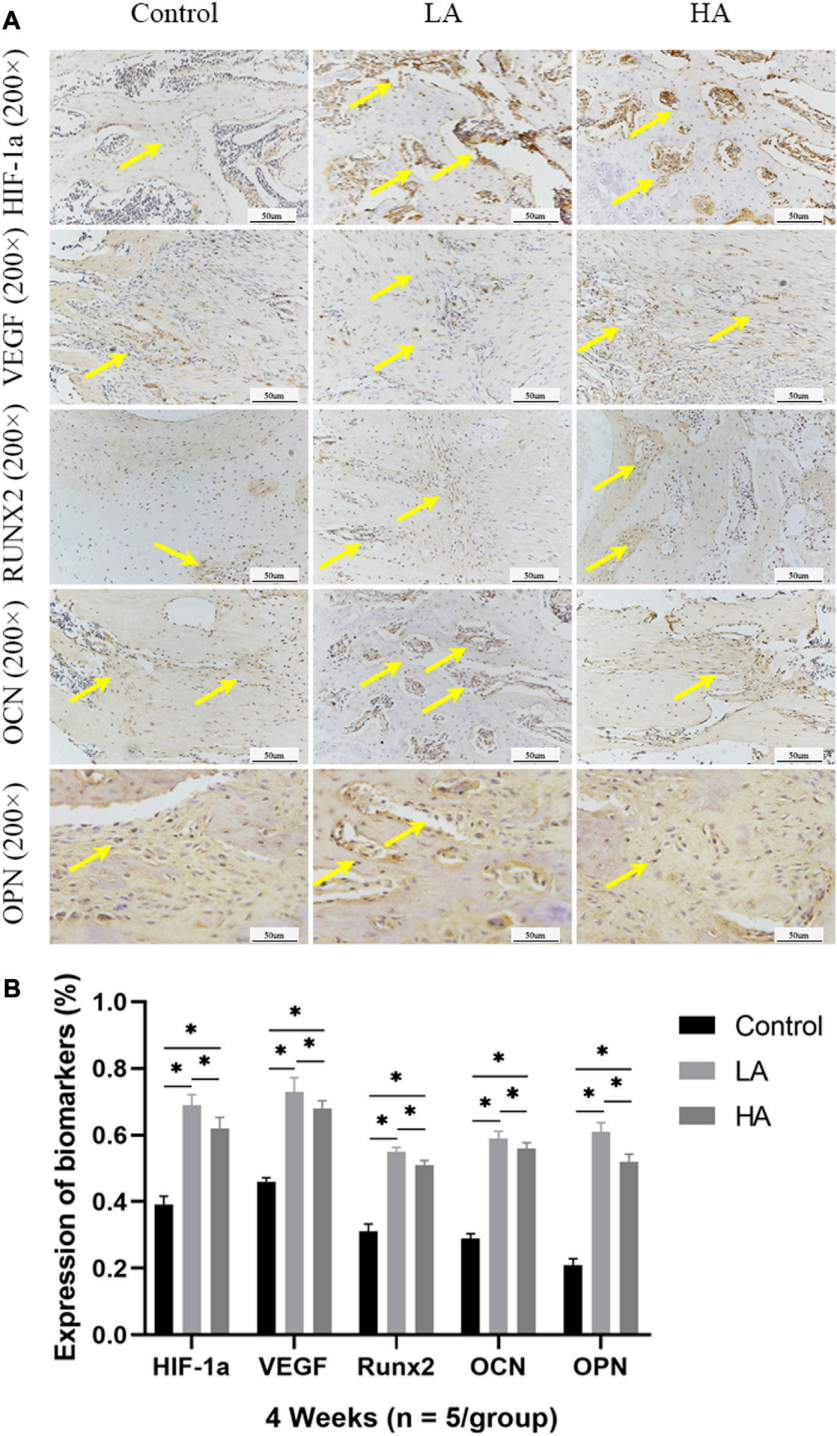
Immunohistochemistry images (high-magnification 200 ×) in the three groups at 4 weeks of consolidation phase **(A)** At the 4 weeks of consolidation phase, as osteoblasts and chondroblasts differentiate into mature osteocytes and mineralization progresses, the expression of angiogenic (HIF-1α, VEGF) and osteogenesis-related proteins (RUNX2, OCN, and OPN) progressively decreased in all three groups. Notably, in Group LA, the positive staining area or cells (yellow arrows) remained predominantly concentrated in the central region of the distraction area. However, in Group HA and the Control group, they were primarily observed at the edge of the distraction area. **(B)** The semi-quantitative measurements showed that the above biomarkers were highly expressed in Group LA, compared to Group HA and the Control (*p < 0.05).

**FIGURE 7:**
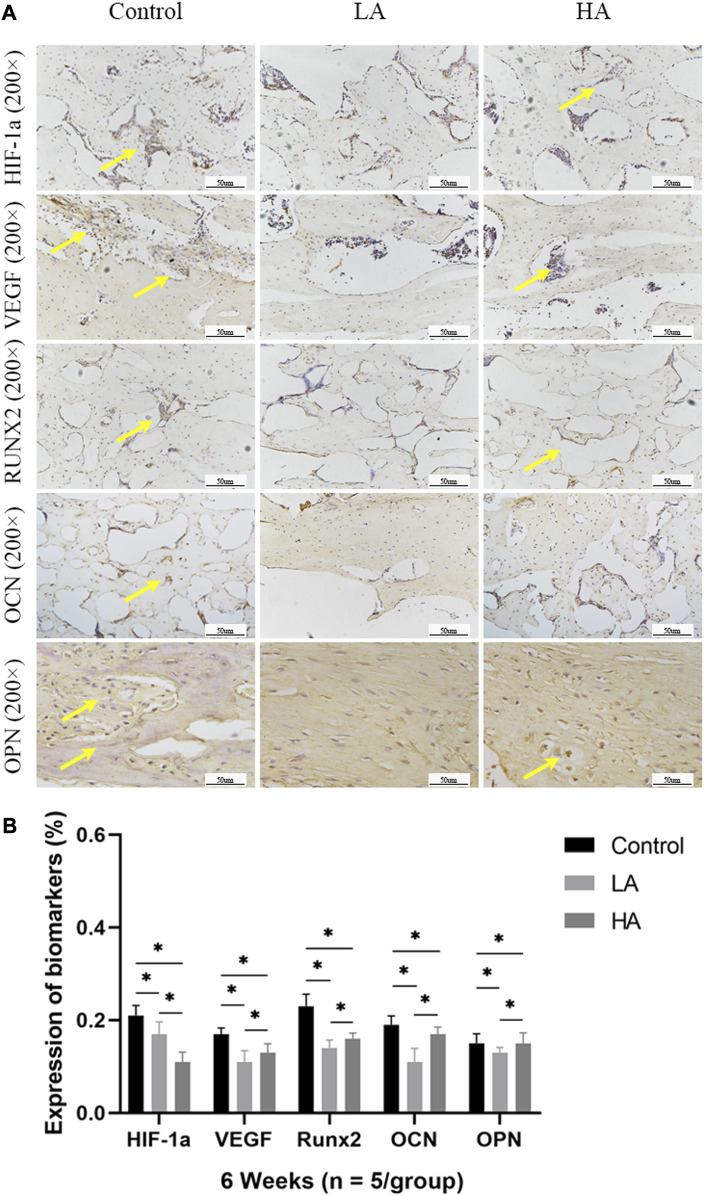
Immunohistochemistry images (high-magnification 200 ×) in the three groups at 6 weeks of consolidation phase **(A)** At the 6 weeks of consolidation phase, the positive staining area or cells (yellow arrows) in the Control were primarily concentrated in the central region of the distraction area. However, there were only a few positively stained areas or cells in Group LA and Group HA, which can be attributed to the recanalization of the medullary cavity and bone remodeling. **(B)** The semi-quantitative measurements revealed that the expression levels of HIF-1α, VEGF, RUNX2, OCN, and OPN were higher in the Control group compared to both Group LA and Group HA (*p < 0.05).

At 2 weeks of consolidation phase, the positive staining area or cells in Group LA were predominantly localized in the central region of the distraction area, whereas in Group HA and the Control group, they were mainly observed at the edge of the distraction area. The results of the semi-quantitative analysis showed that the expression level of HIF-1α, VEGF, RUNX2, OCN, and OPN (Figure 5) was higher in Group LA (76.8% ± 9.5%, 82.45% ± 9.1%, 54.89% ± 1.58%, 61.56% ± 3.12%, and 57.99% ± 4.57%) at 2 weeks of consolidation phase, compared with Group HA (63.79% ± 8.5%, 69.49% ± 3.36%, 39.64% ± 1.29%, 50.17% ± 2.13%, and 45.56% ± 2.14%) (Group LA vs. Group HA, *p* = 0.023) and the Control (34.08% ± 3.72%, 47.56% ± 2.56%, 26.94% ± 4.05%, 26.61% ± 10.68%, and 22.78% ± 1.87%) (Group LA vs. Control, *p* = 0.008; Group HA vs. Control, *p* = 0.016). These findings indicated that the AT protocol implemented in Group LA significantly contributed to the enhanced expression of angiogenic and osteogenesis-related factors and proteins in the distraction area. At the 4 weeks of consolidation phase, as osteoblasts and chondroblasts differentiate into mature osteocytes and mineralization progresses, the expression of angiogenic (HIF-1α, VEGF) and osteogenesis-related proteins (RUNX2, OCN, and OPN) progressively decreased in all three groups. Notably, the positive staining area or cells in Group LA remained predominantly concentrated in the central region of the distraction area. However, they were primarily observed at the edge of the distraction area in Group HA and the Control group. Semi-quantitative analysis of the expression level of HIF-1α, VEGF, RUNX2, OCN, and OPN (Figure 6) further presented a decreasing trend in Group LA (67.61% ± 4.04%, 73.19% ± 4.66%, 54.79% ± 1.33%, 59.74% ± 2.68%, and 59.27% ± 2.54%), but still higher than those in Group HA (62.37% ± 2.01%, 67.35% ± 0.36%, 51.57% ± 0.99%, 55.47% ± 1.21%, and 52.5% ± 1.69%) (Group LA vs. Group HA, *p* = 0.011) and the Control (40.5% ± 2.49%, 45.65% ± 0.63%, 30.38% ± 2.48%, 29.03% ± 0.93%, and 22.28% ± 1.31%) (Group LA vs. Control, *p* = 0.007; Group HA vs. Control, *p* = 0.027). At the 6 weeks of consolidation phase, the positive staining area or cells in the Control were primarily concentrated in the central region of the distraction area. However, in Group LA and Group HA, there were only a few positively stained areas or cells, which can be attributed to the recanalization of the medullary cavity and bone remodeling. The semi-quantitative measurements revealed that the expression level of the above biomarkers (Figure 7) was lower in Group LA (14.48% ± 3.35%, 11.46% ± 2.45%, 13.99% ± 1.69%, 10.25% ± 1.44%, and 12.64% ± 0.83%), compared with Group HA (12.76% ± 0.74%, 13.27% ± 1.15%, 15.65% ± 1.5%, 15.1% ± 1.32%, and 15.89% ± 1.59%) (Group LA vs. Group HA, *p* = 0.025) and the Control (21.12% ± 2.1%, 16.67% ± 1.32%, 22.96% ± 2.67%, 19.97% ± 1.93%, and 15.87% ± 2.12%) (Group LA vs. Control, *p* = 0.037; Group HA vs. Control, *p* = 0.042).

## Discussion

The quality of bone regeneration in the distraction area relies on several factors, including osteoinductivity, osseointegration, angiogenesis of the callus, and the biomechanical environment (Inan et al.,2005; Borzunov,2012; Bezstarosti et al.,2021; Huang et al.,2022). Published studies (Ai-Aql et al., 2008; Sojo et al.,2005; Morgan et al.,2012; Pacicca et al.,2003) have shown that DO triggers a more intense angiogenic response compared to normal fracture healing, with neovascularization growing in the same direction as the primary mineralization front. In our study, we observed distinct transparent bands representing the primary mineralization front in the distraction area, which were more prominent and widespread in Group LA. Additionally, active angiogenesis and osteogenesis were evident in Group LA compared to the Control and Group HA. In our opinion, the application of AT during the distraction phase can synergize with DO to increase the occurrence of a hypoxic environment in the distraction area, leading to the activation of the hypoxia-inducible factor pathway, increased expression of HIF-1α and VEGF, and upregulation of osteogenic-angiogenic coupling, thereby enhancing the process of angiogenesis. However, AT with high amplitude will result in less expression of HIF-1α and VEGF.

The key to the successful application of AT is maintaining a dynamic mechanical force that should closely mimic the natural process of bone formation. However, determining the optimal combination of amplitude and interval for maintaining an appropriate daily distraction rate to promote bone regeneration is still a challenge. Xu et al. (Xu et al.,2018) suggested that applying AT during the middle phase of consolidation, with 3.5 days of compression followed by 3.5 days of distraction at a rate of 0.5 mm/12 h, could significantly promote bone regeneration. Furthermore, Liu et al. (Liu et al.,2022) demonstrated that moderately increasing the amplitude and slowing down the distraction rate using a 5-day compression and 5-day distraction cycle at a rate of 0.25 mm/12 h for 10 days was effective in enhancing bone formation. In this study, we implemented an AT protocol involving different amplitudes of distraction and compression with a 12-h interval, resulting in a total daily distraction rate of 0.5 mm/day. Digital radiographic results revealed that the bone quality in terms of regenerated bone volume and continuity in Group LA and Group HA were improved compared to the Control group at the 6 weeks of consolidation phase. Moreover, quantitative analysis based on micro-CT results and mechanical properties of ROI demonstrated that the AT protocol employed in Group LA was more effective in promoting bone regeneration and remodeling than the other two groups. It was also observed that the positive effects of AT on osteogenesis and bone remodeling were diminished when a larger amplitude of distraction and compression was applied, as evidenced in Group HA. Therefore, we concluded that an AT protocol proved to be advantageous for bone regeneration.

As widely accepted, osteogenesis and angiogenesis processes are closely interconnected during bone regeneration and remodeling, with HIF playing a crucial role in regulating this osteogenic-angiogenic coupling (Pacicca et al., 2003; Schipani et al.,2009; Xu et al.,2018). Furthermore, bone marrow mesenchymal stem cells release various osteogenic factors, including RUNX2, OCN, and OPN, during the osteogenic differentiation process of bone regeneration, thereby promoting the maturation of osteoblasts (Wang et al.,2007; Jia et al.,2019; Komori,2020). In our study, the expression levels of angiogenic factors (HIF-1α and VEGF) and osteogenic biomarkers (RUNX2, OCN and OPN) were observed to be significantly higher at 2, 4 and 6 weeks of consolidation phase in both Group LA and Group HA, when compared to the Control group. However, as the cartilage-like tissues and callus tissues gradually transformed into mature bone tissue, the expression levels of these biomarkers gradually decreased. Therefore, it can be deduced that the application of AT with low amplitude during the distraction phase can create a favorable mechanical environment. This environment, in turn, can promote the process of angiogenesis and chondrogenesis in the callus tissues located in the distraction area. As a result, it can promote the overall process of bone regeneration and remodeling.

There were limitations existed in this study. Firstly, the observation of bone calcium deposition in the distraction area was not possible due to the use of decalcified bone tissue sections. Secondly, conducting dynamic tests to assess the angiogenesis process during DO could provide valuable insights into the mechanism by which additional therapy promoted bone regeneration. Lastly, no *in vitro* experiment was conducted to validate the effects of mechanical forces on osteoblasts. More comprehensive studies involving *in vitro* and *in vivo* investigations are necessary to further elucidate the molecular mechanisms underlying the application of AT during the distraction phase.

## Conclusion

AT with low amplitude applied during the distraction phase can enhance chondrogenesis and bone regeneration by activating the angiogenesis factor pathway and upregulating the expression of osteogenic-related biomarkers such as VEGF, HIF-1α, RUNX2, OCN, and OPN. This stimulation promotes bone regeneration and remodeling. Further, AT during DO is also hoped to be a simple and practical method to perform in clinical practice to reduce the treatment time and prevent the occurrence of poor bone regeneration, delayed union or nonunion.

## Data Availability

The original contributions presented in the study are included in the article/Supplementary Material, further inquiries can be directed to the corresponding author.
