# Supplementary figures and images for: The accordion technique enhances bone regeneration via angiogenesis factor in a rat distraction osteogenesis model
Source: Front Physiol. 2023 Sep 8;14:1259567. doi: 10.3389/fphys.2023.1259567 (PMC10514895; doi:10.3389/fphys.2023.1259567)

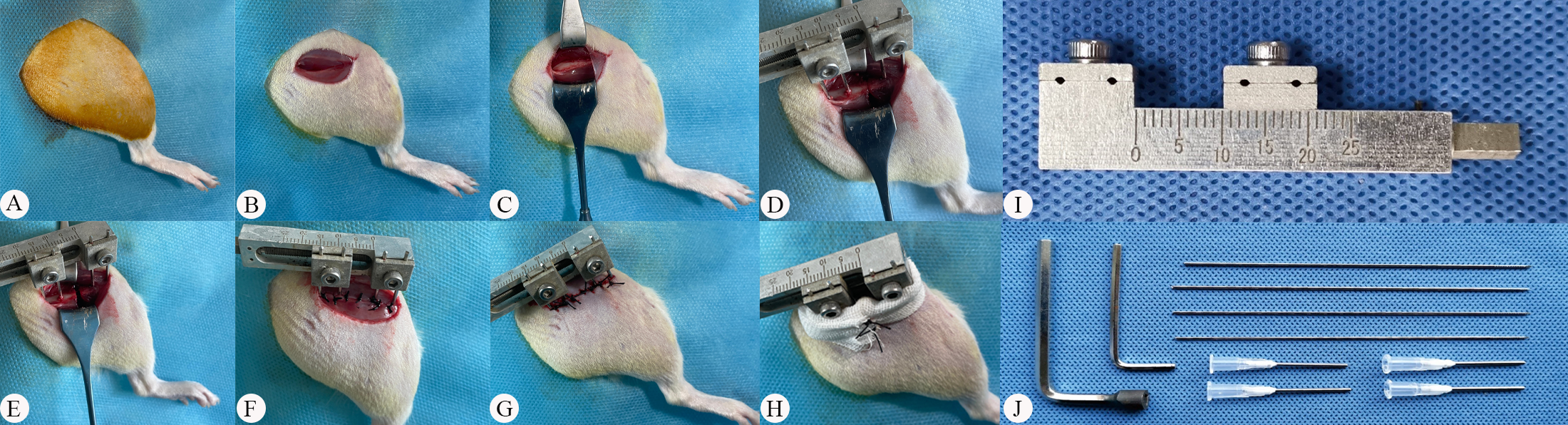

Supplement: Supplementary file 1 [file Image1.TIF]
