# Supplementary material for: The accordion technique enhances bone regeneration via angiogenesis factor in a rat distraction osteogenesis model
Source: Front Physiol. 2023 Sep 8;14:1259567. doi: 10.3389/fphys.2023.1259567 (PMC10514895; doi:10.3389/fphys.2023.1259567)
Supplement: Supplementary file 2 [file DataSheet1.docx]

**Supplementary Data**

The modulus of elasticity (E-modulus) was calculated as follows:

(1)$E=\frac{L^{3}}{48I}\left(\frac{\Delta F}{\Delta\delta}\right)$

*L* (the fulcrum span) = 18 mm, *F* was a deflection, and *I* was a moment of inertia of fracture surface caused by the three-point bending mechanical test.

The energy to failure was calculated as follows:

(2)$W=\int_{0}^{\delta_{0}} F\text{ d}\delta$

Work-of-fracture for the three-point bending test was calculated as the area under the load-displacement curve divided by the specimen's cross-sectional area, and reported in units of J/m^2^. *F* was a deflection.

(3)$G=\frac{W}{A}=\frac{\int_{0}^{\delta_{0}} F\text{ d}\delta}{\pi\left(a_{\mathrm{out}}b_{\mathrm{out}}-a_{\mathrm{in}}b_{\mathrm{in}}\right)}$

*a_out_* (long half axes of the outer ring of collected femur) = 2.25 mm, *b_out_* (short half axes of the outer ring of collected femur) = 1.75 mm, the long and short half axes of the inner ring were *a_in_* (long half axes of the inner ring of the collected femur) = 1.5 mm, *b_in_* (short half axes of the inner ring of collected femur) = 1 mm, *A* was the cross-sectional area of the fracture, and *F* was a deflection.

**
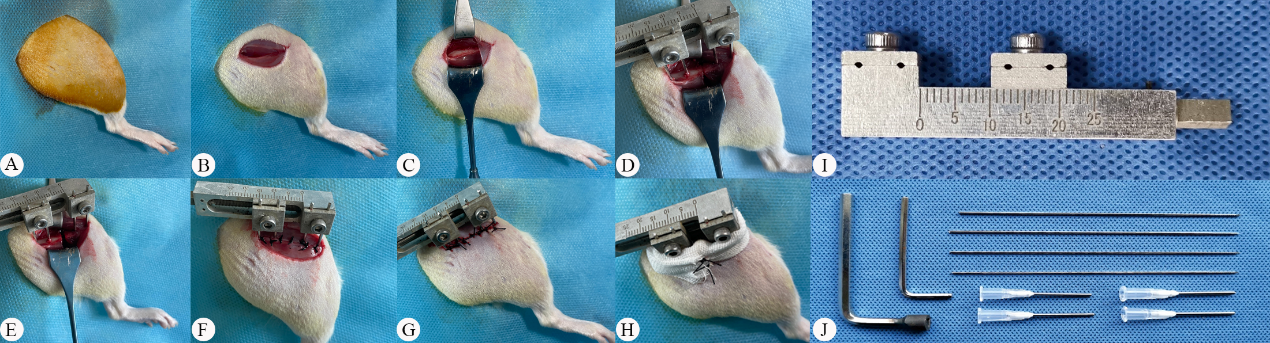
**

**Fig. S1 Surgical procedures of right femoral DO in the rat.**
